# Tumor cell intrinsic dsRNA innate immune response triggered by PARP inhibitor is compromised in BRCA1-deficient breast cancer by repressing IRF3

**DOI:** 10.1093/procel/pwaf104

**Published:** 2026-01-10

**Authors:** Cuiting Zhang, Jing-Bo Zhou, Josh Haipeng Lei, Irene Ling Ang, Kai Miao, Xiaoling Xu, Terence Chuen Wai Poon, Edwin Cheung, Chu-Xia Deng

**Affiliations:** Cancer Centre, University of Macao, Taipa 999078, Macao, China; Centre for Precision Medicine Research and Training, University of Macao, Taipa 999078, Macao, China; MoE Frontiers Science Center for Precision Oncology, University of Macao, Taipa 999078, Macao, China; Faculty of Health Sciences, University of Macao, Taipa 999078, Macao, China; Cancer Centre, University of Macao, Taipa 999078, Macao, China; Centre for Precision Medicine Research and Training, University of Macao, Taipa 999078, Macao, China; MoE Frontiers Science Center for Precision Oncology, University of Macao, Taipa 999078, Macao, China; Faculty of Health Sciences, University of Macao, Taipa 999078, Macao, China; Cancer Centre, University of Macao, Taipa 999078, Macao, China; Centre for Precision Medicine Research and Training, University of Macao, Taipa 999078, Macao, China; MoE Frontiers Science Center for Precision Oncology, University of Macao, Taipa 999078, Macao, China; Faculty of Health Sciences, University of Macao, Taipa 999078, Macao, China; Cancer Centre, University of Macao, Taipa 999078, Macao, China; Centre for Precision Medicine Research and Training, University of Macao, Taipa 999078, Macao, China; MoE Frontiers Science Center for Precision Oncology, University of Macao, Taipa 999078, Macao, China; Faculty of Health Sciences, University of Macao, Taipa 999078, Macao, China; Pilot Laboratory, University of Macao, Taipa 999078, Macao, China; Institute of Translational Medicine, University of Macao, Taipa 999078, Macao, China; Cancer Centre, University of Macao, Taipa 999078, Macao, China; Centre for Precision Medicine Research and Training, University of Macao, Taipa 999078, Macao, China; MoE Frontiers Science Center for Precision Oncology, University of Macao, Taipa 999078, Macao, China; Faculty of Health Sciences, University of Macao, Taipa 999078, Macao, China; Cancer Centre, University of Macao, Taipa 999078, Macao, China; Centre for Precision Medicine Research and Training, University of Macao, Taipa 999078, Macao, China; MoE Frontiers Science Center for Precision Oncology, University of Macao, Taipa 999078, Macao, China; Faculty of Health Sciences, University of Macao, Taipa 999078, Macao, China; Cancer Centre, University of Macao, Taipa 999078, Macao, China; Centre for Precision Medicine Research and Training, University of Macao, Taipa 999078, Macao, China; MoE Frontiers Science Center for Precision Oncology, University of Macao, Taipa 999078, Macao, China; Faculty of Health Sciences, University of Macao, Taipa 999078, Macao, China; Pilot Laboratory, University of Macao, Taipa 999078, Macao, China; Institute of Translational Medicine, University of Macao, Taipa 999078, Macao, China; Cancer Centre, University of Macao, Taipa 999078, Macao, China; Centre for Precision Medicine Research and Training, University of Macao, Taipa 999078, Macao, China; MoE Frontiers Science Center for Precision Oncology, University of Macao, Taipa 999078, Macao, China; Faculty of Health Sciences, University of Macao, Taipa 999078, Macao, China; Cancer Centre, University of Macao, Taipa 999078, Macao, China; Centre for Precision Medicine Research and Training, University of Macao, Taipa 999078, Macao, China; MoE Frontiers Science Center for Precision Oncology, University of Macao, Taipa 999078, Macao, China; Faculty of Health Sciences, University of Macao, Taipa 999078, Macao, China

**Keywords:** PARP inhibitor, limited proteolysis-mass spectrometry, innate immune response, BRCA1 deficiency, IRF3, poly(I:C)

## Abstract

Poly(ADP-ribose) polymerase 1 (PARP1) inhibition represents a promising targeted therapy for BRCA-deficient cancer patients based on the synthetic lethality theory. Recent evidence shows that the efficacy of DNA damage drugs depends on two aspects: DNA repair signaling and immune response. Applying a functional proteomics approach, we find that the function of the spliceosome is perturbed by PARP inhibitors via enhancing interaction between PARP1 and SF3B1, a key factor of the spliceosome. We demonstrate that differential alternative spliced mRNA and accumulation of double-stranded RNA (dsRNA) are induced by perturbation of the spliceosome upon PARP inhibitor treatment, resulting in triggering dsRNA antiviral mimicry innate immune response. Moreover, we identify a novel function of BRCA1, through which BRCA1 regulates innate immune response, leading to compromising of the innate immune signaling by downregulation of IRF3 in BRCA1-deficient breast cancer cells, which reduces the sensitivity to PARP inhibitors and causes intrinsic resistance. Polyinosinic–polycytidylic acid (poly(I:C)) is a dsRNA synthetic analog sensitizing PARP inhibitors through further triggering dsRNA signaling. Finally, we show that the combination of PARP inhibitors and poly(I:C) enhances anti-tumor efficiency *in vivo*. Overall, our study reveals that BRCA1 deficiency impedes tumor cell intrinsic innate immune response, inducing intrinsic resistance to PARP inhibitors that can be overcome when poly(I:C) is combined.

## Introduction

Breast cancer is the most widespread cancer in women globally. In 2022, there were 2,296,840 newly diagnosed cases and 666,103 dead cases, accounting for 15.4% of cancer deaths in women (Bray et al., 2024). Inherited breast cancer cases are about 10%. Approximately 60% of inherited breast cancer patients have breast cancer-associated gene 1 (BRCA1) or BRCA2 mutations (Peleg Hasson et al., 2020). Up to 87% of BRCA1 mutation carriers will get breast cancer by age 70, while it is 8% in the general population (Jimenez-Sainz and Jensen, 2021). The BRCA1-associated genome surveillance complex is central to DNA repair for the maintenance of genome stability. BRCA1 is well known for its function in homologous recombination (HR), a high-fidelity DNA double-strand break (DSB) repair mechanism. HR appears to be the most important DSB repair pathway for protecting the integrity of the genome, whereas other DSB repair mechanisms, including nonhomologous DNA end joining (NHEJ) and microhomology-mediated end joining (MMEJ), are error-prone and result in genomic aberrations such as radial chromosomes, deletions, and translocations, which cause cell death and cancers (Chen et al., 2020; Tarsounas and Sung, 2020; Xu et al., 1999a, 1999b, 2001).

Poly(ADP-ribose) polymerase 1 (PARP1) inhibitors (PARPi) are used as a targeted therapy for BRCA-deficient cancer patients. They cause synthetic lethality in BRCA-deficient tumors because DNA damage repair in BRCA-deficient cells relies on PARP-mediated error-prone pathways, and DSBs cannot be repaired properly in HR-deficient cells upon PARPi treatment (Bryant et al., 2005; Farmer et al., 2005). PARP1 is a factor in single-strand DNA break (SSB) repair, base-excision repair (BER), and NHEJ via post-translational poly ADP-ribosylation (PARylation) itself and its target proteins (El-Khamisy et al., 2003; Jungmichel et al., 2013). The first approved PARPi, ­olaparib, has been used for BRCA-mutated breast cancer, platinum-responsive advanced ovarian cancer, BRCA-mutated pancreatic cancer, and HR-deficient prostate cancer. PARP1 also involves other biological processes such as inflammation, epigenetic events, and modification of chromatin structure and histones (Guastafierro et al., 2008; Hassa et al., 2005).

Besides, increasing evidence indicates that DNA-damaging drugs and radiation therapy activate the innate immune response. DNA fragments, also known as double-stranded DNA (dsDNA), are generated from damaged DNA, mimicking DNA virus infection. After releasing into the cytoplasm, dsDNA binds to cytosolic DNA-cyclic GMP/AMP synthase complex (cGAS) and activates the stimulator of interferon genes (STING). cGAS-STING pathway then induces interferon (IFN) signaling through phosphorylation of TANK-binding protein kinase 1 (TBK1) and interferon regulatory factor 3 (IRF3) (Ding et al., 2018; Pantelidou et al., 2019; Shen et al., 2019). Moreover, the combination of STING agonist and PARPi enhances the anti-tumor efficiency of BRCA1-deficient breast cancer (Wang et al., 2022). However, whether BRCA1 is involved in innate immune response is still unclear. Another mechanism of cellular antiviral response is mitochondrial antiviral signaling protein (MAVS)-mediated dsRNA signaling. Previous studies show that some DNA damage agents activate dsRNA antiviral mimicry response via accumulation of cytosolic dsRNA (Chiappinelli et al., 2015; Guo et al., 2022; Patel et al., 2022). For instance, WEE1 inhibitor triggers dsRNA antiviral mimicry response in cGAS/STING-deficient cells (Guo et al., 2022). Combination of PARPi with euchromatic histone lysine methyltransferases 1 and 2 (EHMT1/2) inhibitor further increases accumulation of dsRNA and diminishes therapy-resistant ovarian cancer growth (Nguyen et al., 2024). Altogether, these observations suggest that BRCA1 and PARP1 might be involved in dsRNA formation and innate immune response, yet their potential connections and functions in these processes remain elusive.

To investigate this, we performed functional proteomics, limited proteolysis–mass spectrometry (LiP-MS) to monitor functionally changed proteins in breast cancer cells upon PARPi olaparib treatment (Cappelletti et al., 2021; Schopper et al., 2017). We discover that PARPi dysregulates the function of the spliceosome. Moreover, we show that perturbation of the spliceosome by PARPi causes differential alternative spliced mRNA and accumulation of dsRNA, which triggers dsRNA antiviral mimicry innate immune response. We show that BRCA1 regulates innate immune response, as indicated by higher levels of dsRNA signaling in BRCA1 wild-type breast cancer cells than in BRCA1-deficient cells. Furthermore, the compromised innate immune response in BRCA1-deficient breast cancer cells induces resistance to PARPi. Finally, we demonstrate that dsRNA analog polyinosinic–polycytidylic acid (poly(I:C)) sensitizes PARPi through further triggering this signaling and significantly enhances anti-tumor effect.

## Results

### PARPi dysregulates the function of the spliceosome, revealed by LiP-MS

**Figure 1.**
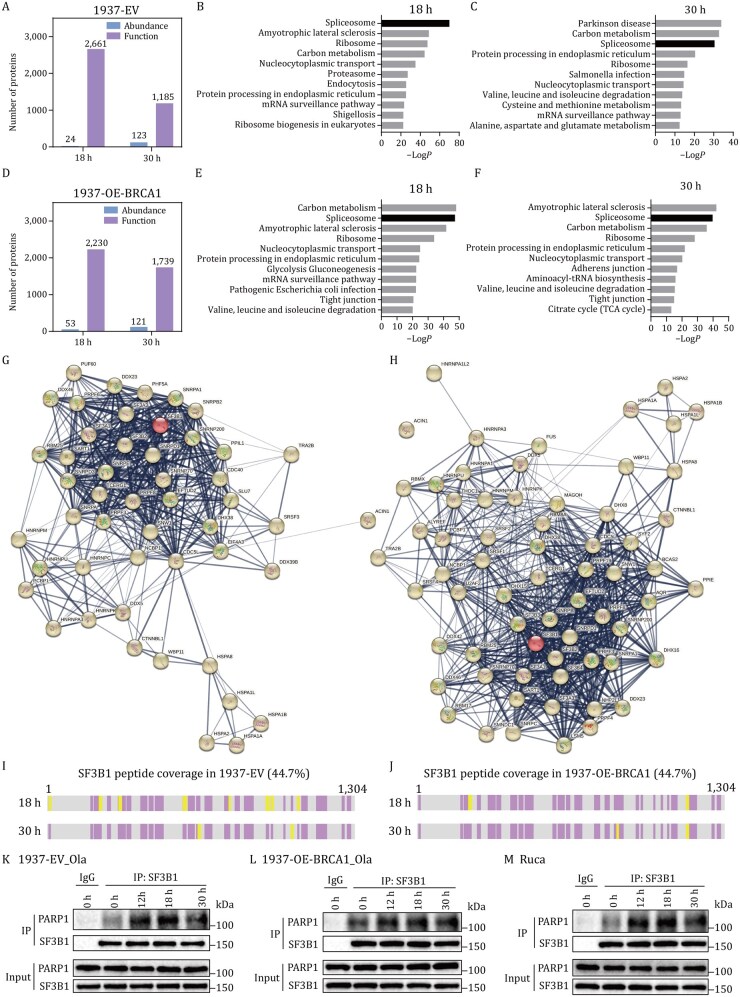
**LiP-MS identifies the function of the spliceosome is dysregulated by PARPi**. (A and D) The number of proteins with significantly abundance change (blue) (|log_2_FC| > 0.5, adjusted *P*-value < 0.05) and function changes (purple) (|log_2_FC| > 2, adjusted *P*-value < 0.01) in HCC1937-EV and HCC1937-OE-BRCA1 subjected to 10 µmol/L olaparib treatment for 18 h or 30 h. (B, C, E, and F) KEGG enrichment analysis of significantly functionally changed proteins in A and D. (G and H) STRING protein–protein network analysis among the protein list of the spliceosome enriched in C and F. (I and J) Peptide coverage of SF3B1 from N to C terminus. Yellow indicated the peptides with significant change of intensity (|log_2_FC| > 2, adjusted *P*-value < 0.01). Peptides that did not have a significant change in intensity are shown as purple bars. Gray indicated peptides that were not detected. (K and L) SF3B1 coimmunoprecipitation assays in HCC1937-EV and HCC1937-OE-BRCA1 upon 10 µmol/L olaparib treatment for 12 h, 18 h, or 30 h. (M) SF3B1 coimmunoprecipitation assays in HCC1937 upon 10 µmol/L rucaparib treatment for 12 h, 18 h, or 30 h.

We performed LiP-MS analysis on a pair of congenic cell lines, HCC1937-EV (a BRCA1 mutated cell line HCC1937 carrying an empty vector) and HCC1937-OE-BRCA1, upon PARPi olaparib treatment (Fig. S1A) to identify proteins with functional and abundance changes. LiP-MS identifies proteins with structural and abundance changes, and these structural changes reflect protein function state, so-called functional proteomics (Cappelletti et al., 2021; Schopper et al., 2017). Any transformations that affect the accessibility of proteinase K (PK), a key step of LiP-MS, such as protein–protein interaction, protein–DNA interaction, binding with small molecules, post-translational modification, protein cleavage, and protein aggregation, can lead to intensity changes of peptides (Cappelletti et al., 2021; Schopper et al., 2017). By comparing experimental conditions, we can pinpoint proteins whose function and level vary, providing insights into the biological processes and pathways affected (Cappelletti et al., 2021; Schopper et al., 2017). Similar to the previous publication, we found a slightly smaller band in HCC1937-EV due to expression of a truncated BRCA1 protein that is caused by the 5382insC mutation, while HCC1937-OE-BRCA1 expressed both truncated BRCA1 and transfected wild-type BRCA1 (Fig. S1B) (Scully et al., 1999). Overall, we detected 2,661 and 1,185 functionally changed proteins at 18 h and 30 h after the treatment in HCC1937-EV cells while 2,230 and 1,739 in HCC1937-OE-BRCA1 cells (Fig. 1A and 1D). The results showed that fewer proteins exhibited changes in abundance, while a significantly larger number of proteins had functional changes. This observation aligns with findings from previous studies on other targets (Cappelletti et al., 2021; Schopper et al., 2017). Then, we categorized the functionally changed proteins according to their biological processes. 172 and 153 DNA repair-related proteins of HCC1937-EV and HCC1937-OE-BRCA1 were enriched due to DNA damage response (DDR) after PARPi treatment, as expected (Fig. S1C and S1D). As LiP-MS can identify proteins that bind with small molecules, we found that PARP1 was captured by LiP-MS because of structural changes of PARP1 when binding with olaparib, which will further affect accessibility of PK, a critical step in LiP-MS (Ogden et al., 2021). Therefore, multiple LiP peptides were shown for PARP1 in the LiP-MS results (Fig. S2A–D). In addition, we performed protein aggregation assay upon PARPi treatment using the PROTEOSTAT Protein Aggregation Kit. After olaparib treatment on HCC1937 cells, the fluorescence intensity of protein aggregation was significantly increased (Fig. S2E). The result suggests that the cells after PARPi treatment might also induce protein aggregation and proteotoxic stress (Li et al., 2025; Shao et al., 2020, 2025).

One of the top-enriched signaling pathways that appeared in both HCC1937-EV and HCC1937-OE-BRCA1 cells upon PARPi treatment was the spliceosome (Fig. 1B, 1C, 1E and 1F). The spliceosome plays an important role in mRNA splicing that removes introns from mRNA precursors. It is a fundamental mechanism for the expression of most genes. Scientists have developed small molecules like SF3B1 inhibitors to perturb the function of the spliceosome due to its relationship with cancer progression and recurrence (Bowling et al., 2021; Lu et al., 2021). Among all enriched spliceosome factors (Figs. 1G, 1H, S3A and S3B), SF3B1 showed most significantly changed LiP peptides (Fig. S3C–F) and it is a key factor with the highest heterozygous somatic mutations frequency in cancer patients while highly expression of SF3B1 also relates to cancer aggressiveness and poor survival rates, indicating SF3B1 homeostasis is critical (Han et al., 2022; López-Cánovas et al., 2021). SF3B1 mutated cells showed a significantly synthetic lethal effect with PARPi through drug sensitivity screening, indicating functional crosstalk between SF3B1 and PARP1 (Bland et al., 2023). We further checked and mapped LiP peptides of SF3B1 (Figs. 1I, 1J and S4A–D). Some LiP peptides were shown for SF3B1. These data suggest the function of the spliceosome is dysregulated by PARPi. Previous study showed that regulation of mRNA splicing by PARP1 depends on interaction between PARP1 and SF3B1 but not PARylation enzyme activity of PARP1 (Matveeva et al., 2016). To further confirm dysregulation of the spliceosome upon PARPi treatment by affecting the interaction between PARP1 and SF3B1, we performed an SF3B1 co-immunoprecipitation assay. With PARPi treatment including olaparib and rucaparib, immunoprecipitation of SF3B1 pulled down more PARP1 (Figs. 1K–M and S4E–G), indicating that PARPi disturbs function of the spliceosome at least partially by enhancing interaction between PARP1 and SF3B1.

### PARPi perturbs the spliceosome, causing the accumulation of dsRNA and differential alternative spliced mRNA

**Figure 2.**
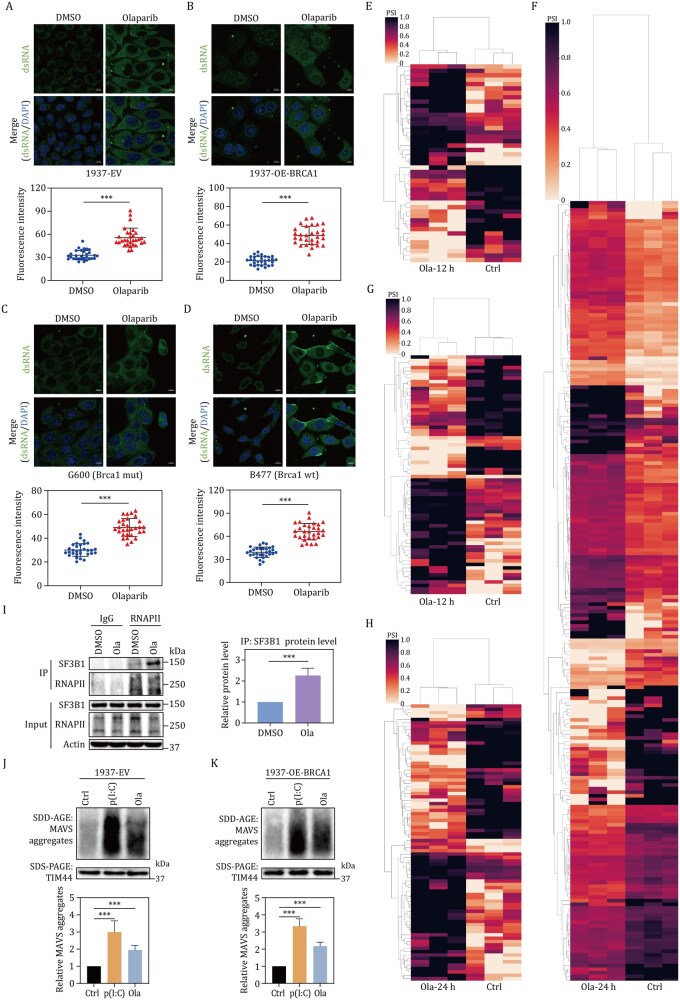
**PARPi perturbs the spliceosome thus causes accumulation of dsRNA and differential alternative spliced mRNA**. (A–D) Anti-dsRNA (J2) immunofluorescence (IF) staining in HCC1937-EV, HCC1937-OE-BRCA1, G600, and B477 with or without 10 µmol/L olaparib treatment for 12 h. Scale bars, 10 µm. Fluorescence intensities were quantified by ZEISS ZEN 2 software. (E–H) Heatmap of differential alternative splicing events in HCC1937-EV (E and F), HCC1937-OE-BRCA1 (G and H), with 10 µmol/L olaparib treatment for 12 h or 24 h. (I) RNAPII coimmunoprecipitation assay in HCC1937 with or without 10 µmol/L olaparib treatment. Coimmunoprecipitated SF3B1 protein levels were quantified and plotted relative to the control group. (J and K) MAVS aggregation on mitochondria analyzed by SDD-AGE in HCC1937-EV and HCC1937-OE-BRCA1 upon olaparib or poly(I:C) treatment. MAVS aggregates levels were quantified and plotted relative to the control group. Data are presented as mean ± SD. ns, not significant; *P < 0.05; **P < 0.01; ***P < 0.001 (two-way ANOVA, *n *= 3).

Dysregulation of the spliceosome by spliceosome-targeted therapies like SF3B1 inhibitors, RBM39 degrader, and type I PRMT inhibition triggers antiviral innate immune response through differential alternative spliced mRNA and accumulation of dsRNA (Bowling et al., 2021; Lu et al., 2021). To further validate dysregulation of splicing by PARPi, we conducted immunofluorescence staining of dsRNA using a specific antibody (mouse monoclonal J2 antibody) in two pairs of breast cancer cell lines, HCC1937-EV and HCC1937-OE-BRCA1, and G600 (Brca1 mutant) and B477 (Brca1 wild type) (Xu et al., 2019). The intensity of immunofluorescence signal was significantly increased after PARPi treatment in each cell line. The results indicated PARPi induced significant accumulation of dsRNA in cytoplasm (Fig. 2A–D), consistent with earlier studies that accumulation of cytoplasmic dsRNA was induced by perturbation of the spliceosome (Bowling et al., 2021). We then detected differential alternative splicing events. To do this, we performed RNA-seq on both HCC1937-EV and HCC1937-OE-BRCA1 cells with PARPi treatment, and we analyzed differential alternative splicing events by a computational tool, MATS, that was developed by Xing lab (Shen et al., 2014). MATS calculates percent spliced in (PSI) values for each exon from reads of RNA-seq to indicate splicing variants (Shen et al., 2014). It applies a likelihood-ratio test to compare the mean PSI values between two sample groups to determine statistical significance (Shen et al., 2014). Totally, we identified 261 and 150 differential alternative splicing events, respectively (Fig. 2E–H). These events were classified into five subtypes, including retained intron (RI), alternative 3′ splice sites (A3SS), alternative 5′ splice sites (A5SS), skipped exon (SE), and mutually exclusive exons (MXE). About 20% of events were comprised of A3SS, A5SS, and RI, while the remaining were SE and MXE (Fig. S5A and S5B). Interestingly, MXE was drastically increased in 1937-EV after olaparib treatment for 24 h. In addition, we validated the differential alternative splicing event SE 31 of NCOR1 that was induced upon olaparib treatment by PCR analysis, while unchanged expression level and size of NCoR1 protein were observed through western blot by our existing antibody (Fig. S5C and S5D). Collectively, these data support that PARPi dysregulates function of the spliceosome, which causes accumulation of dsRNA and differential alternative spliced mRNA.

Additionally, the SF3B1 spliceosome complex binds with RNA polymerase II (RNAPII) to splice mRNA properly when RNAPII mediates transcription (Panzeri et al., 2023). To further explore the underlying mechanism of how PARPi affects splicing, we hypothesized interaction between SF3B1 and RNAPII was altered by PARPi treatment. To test the possibility, we immunoprecipitated RNAPII in HCC1937 after PARPi treatment and detected coimmunoprecipitated SF3B1. Our results showed that SF3B1 was coimmunoprecipitated more by immunoprecipitated RNAPII in PARPi-treated cells than in the control group (Fig. 2I). Notably, SF3B1 homeostasis is critical for mRNA splicing (Han et al., 2022; López-Cánovas et al., 2021). Together, these results indicate that PARPi dysregulates splicing through enhancing interaction between RNAPII and SF3B1, besides interaction between PARP1 and SF3B1, resulting in differential alternative spliced mRNA and accumulation of dsRNA.

Accumulated dsRNA will activate MAVS and then induce antiviral mimicry innate immune response. We then asked whether PARPi activates the MAVS-mediated antiviral mimicry signaling pathway. As activated MAVS aggregates on the mitochondria, we conducted semi-denaturing detergent agarose gel electrophoresis (SDD-AGE) of isolated mitochondria upon PARPi treatment. As shown in Fig. 2J and 2K, PARPi caused aggregation of MAVS on the mitochondria in both HCC1937-EV and HCC1937-OE-BRCA1 cells. Synthetic analog of dsRNA, poly(I:C), was used as a positive control and induced a strong aggregation of MAVS on the mitochondria. Collectively, these data indicate PARPi can activate MAVS-mediated dsRNA antiviral mimicry innate immune signaling.

### dsRNA innate immune response triggered by PARPi is dependent on BRCA1

**Figure 3.**
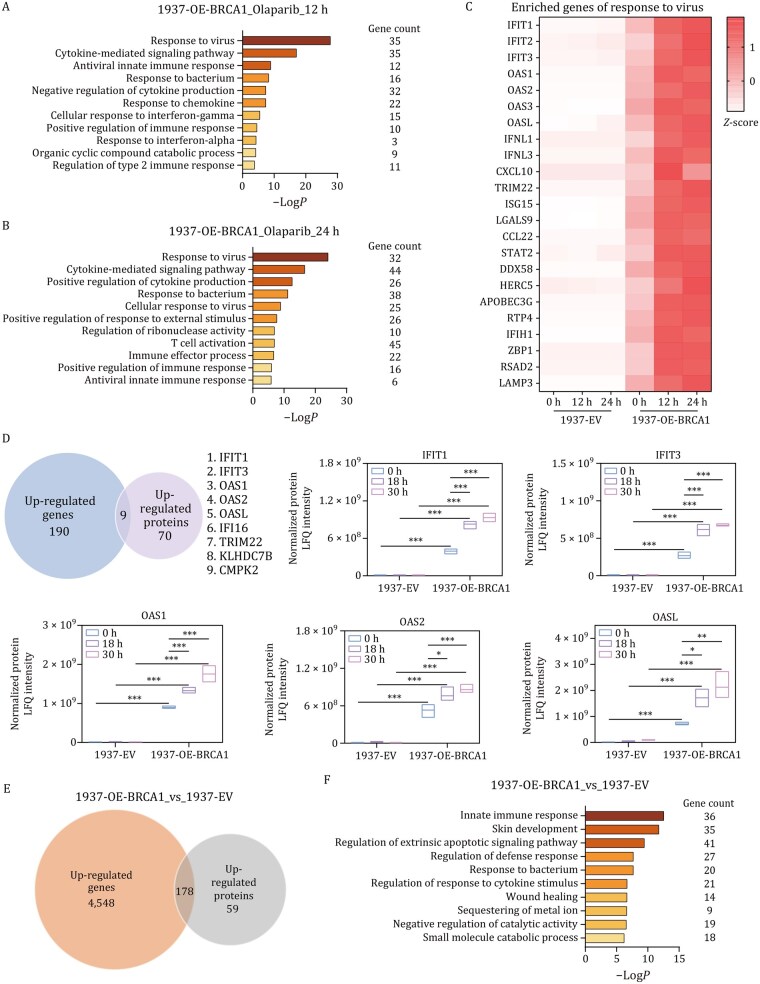
**dsRNA innate immune response triggered by PARPi is dependent on BRCA1**. (A and B) GO biological processes enrichment of upregulated genes from RNA-seq in HCC1937-OE-BRCA1 upon 10 µmol/L olaparib treatment for 12 h or 24 h. (C) Overlapped genes of the highest enriched GO biological process response to the virus in A and B. (D) Overlap of upregulated genes and upregulated proteins from abundance proteomics of HCC1937-OE-BRCA1 with the olaparib treatment group. Normalized protein LFQ intensity of IFIT and OAS proteins was shown in a boxplot. ns, not significant; *P < 0.05; **P < 0.01; ***P < 0.001 (two-way ANOVA, *n *= 3). (E) Venn diagram indicating 178 overlapped up-regulated genes and proteins comparing HCC1937-OE-BRCA1 versus HCC1937-EV. (F) GO biological processes enrichment of overlapped upregulated genes and proteins in (E).

To further study PARPi-induced dsRNA innate immune response, we next examined transcriptome and abundance proteomics. First, we enriched differentially expressed genes of PARPi treatment in HCC1937-EV and HCC1937-OE-BRCA1. The upregulated biological processes in HCC1937-OE-BRCA1 after PARPi treatment were almost all associated with response to virus and antiviral innate immune response (Fig. 3A and 3B). However, strongly enriched biological processes in HCC1937-EV were not immune response-related pathways, but oxidative phosphorylation, positive regulation of reactive oxygen species metabolic process, etc. (Fig. S6A). The common enriched genes of response to virus of different drug treatment durations were shown in Fig. 3C, such as IFN-induced protein with tetratricopeptide repeats (IFIT) and 2′–5′ oligoadenylate synthetase (OAS) family genes. These genes were highly upregulated in HCC1937-OE-BRCA1 compared to HCC1937-EV, especially after PARPi treatment (Figs. 3C and S6B). Meanwhile, we overlapped upregulated genes and upregulated proteins from abundance proteomics of HCC1937-OE-BRCA1 with the PARPi treatment group (Fig. 3D). There were nine overlapped proteins, and most of them were related to dsRNA innate immune response, including IFIT1, IFIT3, OAS1, OAS2, and OASL. IFIT1 coordinating with IFIT3 is reported to protect against RNA viruses by suppressing viral RNA translation (Johnson et al., 2018; Pichlmair et al., 2011). OAS enzymes recognize dsRNA and initiate RNA degradation pathway by activating RNase L (Ibsen et al., 2015; Lee et al., 2022a; Zhu et al., 2014). An abundance of proteins showed time-dependent increase in HCC1937-OE-BRCA1 with PARPi treatment, while only the OASL protein was induced slightly in HCC1937-EV (Figs. 3D and S6C). Notably, the basal level of these proteins varied between HCC1937-EV and HCC1937-OE-BRCA1, which suggested BRCA1 might regulate the dsRNA innate immune response. To confirm this finding, we overlapped upregulated genes and proteins after comparing HCC1937-OE-BRCA1 with HCC1937-EV cells without drug treatment groups (Figs. 3E, S6D and S6E). 178 genes and proteins were identified as overlapping genes and proteins. Enrichment analysis of 178 proteins revealed that the top signaling pathway was innate immune response (Fig. 3F). The GSEA-based approach also indicated that innate immune response and response to virus were enriched in HCC1937-OE-BRCA1 but not HCC1937-EV (Fig. S6F). Taken together, our results indicate that antiviral mimicry innate immune response triggered by PARPi is dependent on BRCA1.

### PARPi-induced dsRNA innate immune response is compromised in BRCA1-deficient breast cancer cells

**Figure 4.**
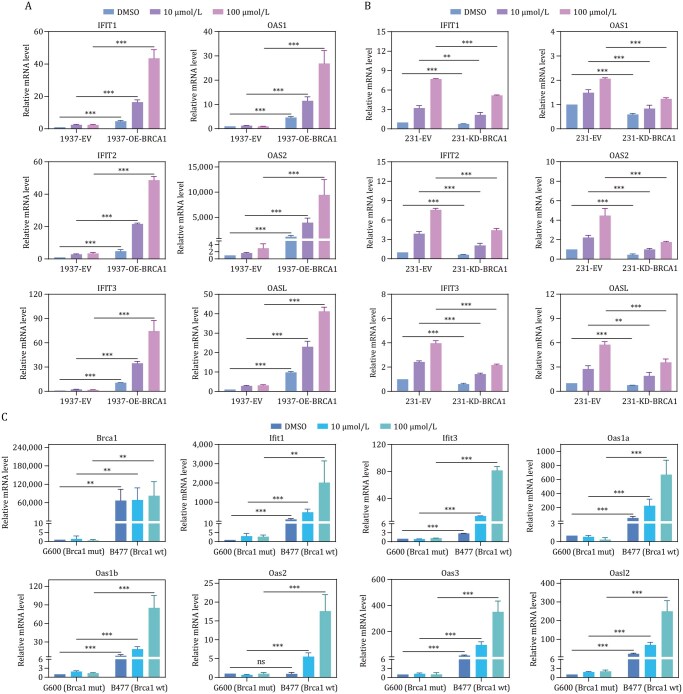
**PARPi-induced dsRNA innate immune response is compromised in BRCA1-deficient breast cancer cells**. (A) Relative mRNA expression of IFIT and OAS genes in HCC1937-EV and HCC1937-OE-BRCA1 upon olaparib treatment. (B) Relative mRNA expression of IFIT and OAS genes in MDA-MB-231 and BRCA1 knockdown MDA-MB-231 cells upon olaparib treatment. (C) Relative mRNA expression of IFIT and OAS genes in G600 (Brca1 mut) and B477 (Brca1 wt) upon olaparib treatment. Data are presented as mean ± SD. ns, not significant; *P < 0.05; **P < 0.01; ***P < 0.001 (two-way ANOVA, *n *= 3).

To further validate whether BRCA1 regulates antiviral mimicry innate immune response, we measured induction of IFIT1, IFIT2, IFIT3, OAS1, OAS2, and OASL in HCC1937 and wild-type BRCA1 overexpressed cells HCC1937-OE-BRCA1 upon 10 µmol/L and 100 µmol/L PARPi olaparib treatment. Consistent with the above findings, overexpression of BRCA1 upregulated these genes, and PARPi treatment induced a significant elevation of these genes in HCC1937-OE-BRCA1 cells compared to HCC1937-EV cells (Fig. 4A). Furthermore, we knocked down BRCA1 in MDA-MB-231 cells and then treated them with PARPi. We observed that IFIT and OAS genes decreased after knockdown of BRCA1 (Fig. 4B). Moreover, PARPi treatment induced the gene expression was also compromised (Fig. 4B). Additionally, we got similar results through a pair of mouse breast cancer cell lines, G600 and B477. Ifit and Oas families’ genes were significantly increased in wild-type Brca1 B477 cells compared to Brca1-deficient G600 cells with or without drug treatment (Fig. 4C). These data indicate that BRCA1 functions in the regulation of dsRNA antiviral mimicry innate immune signaling, and BRCA1 deficiency compromises this signaling.

### IRF3 is downregulated in BRCA1-deficient breast cancer cells, causing resistance to PARPi, which can be overcome by combination with poly(I:C)

**Figure 5.**
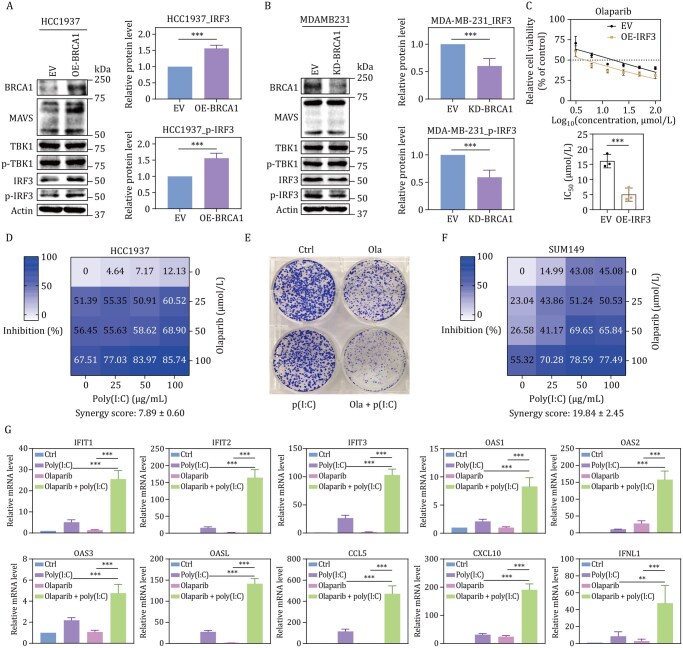
**IRF3 is downregulated in BRCA1-deficient breast cancer cells, causing resistance to PARPi, which can be overcome by combination with poly(I:C)**. (A) Western blot analysis of key factors in dsRNA innate immune signaling, including MAVS, TBK1, phosphorylated TBK1, IRF3, and phosphorylated IRF3 with or without wild-type BRCA1 overexpression in HCC1937. IRF3 and phosphorylated IRF3 protein levels were quantified and normalized to Actin and then plotted relative to control cells. (B) Western blot analysis of key factors in dsRNA innate immune signaling, including MAVS, TBK1, phosphorylated TBK1, IRF3, and phosphorylated IRF3 with or without BRCA1 knockdown in MDA-MB-231. IRF3 and phosphorylated IRF3 protein levels were quantified and normalized to Actin and then plotted relative to control cells. (C) Dose response of olaparib in HCC1937 with or without IRF3 overexpression. IC_50_ of olaparib in HCC1937 was calculated by dose–response curve. (D) Synergy heatmap of combination treatment with olaparib and poly(I:C) in HCC1937. Synergy score was calculated via the SynergyFinder web tool using the HSA method. (E) Colony formation assay of single drug treatment and combination drug of olaparib (10 µmol/L) and poly(I:C) (25 µg/mL) treatment in HCC1937. (F) Synergy heatmap of combination treatment with olaparib and poly(I:C) in SUM149. Synergy score was calculated via the SynergyFinder web tool using the HSA method. (G) Relative mRNA expression of IFIT, OAS, and IFN genes in HCC1937 upon single drug olaparib (10 µmol/L), poly(I:C) (25 µg/mL), or combined drug treatment. Data are presented as mean ± SD. ns, not significant; *P < 0.05; **P < 0.01; ***P < 0.001 (two-way ANOVA, *n *= 3).

To further explore the relationship between BRCA1 and dsRNA innate immune signaling, we asked whether BRCA1 regulates key factors of this signaling, we examined protein level of MAVS, TBK1, phosphorylated TBK1, IRF3 and phosphorylated IRF3 with or without wild type BRCA1 overexpression in HCC1937 (Fig. 5A), as well as BRCA1 knockdown in MDA-MB-231 cells (Fig. 5B). Western blot analysis showed that IRF3 and phosphorylated IRF3 were increased by BRCA1 overexpression and decreased by BRCA1 knockdown indicating BRCA1 upregulates IRF3 while BRCA1 deficiency downregulates IRF3. TBK1 and phosphorylated TBK1 did not have obvious changes after BRCA1 overexpression and knockdown. Because IRF3 decreased in BRCA1-deficient cells, we asked whether the efficiency of PARPi can be improved by further triggering this signaling. To do this, we first examined the drug response after overexpression of IRF3. As shown in Fig. 5C, overexpression of IRF3 induced the drug sensitivity of PARPi. Half-maximal inhibitory concentration (IC_50_) of PARPi olaparib significantly decreased in IRF3 overexpressed cells, indicating a lower level of IRF3 is an intrinsic resistance mechanism of PARPi in BRCA1-deficient breast cancer cells. In addition, dsRNA synthetic analog poly(I:C) is clinically used as a potent immune adjuvant for cancer therapy and an antiviral treatment agent due to its ability to induce antiviral mimicry innate immune response. We then combined poly(I:C) with PARPi to treat BRCA1-deficient breast cancer cells and investigated synergy effects. The synergy score calculated by SynergyFinder of combination treatment in HCC1937 was 7.89, indicating that poly(I:C) and PARPi had additive effect (Fig. 5D). Colony formation assay showed similar results that combination group inhibited colony formation obviously (Fig. 5E). Besides, we tested the combination effect in another human BRCA1-deficient breast cancer cell line SUM149 (Figs. 5F and S7A). The synergy score 19.84 indicated a synergy effect of both drugs, which means PARPi is sensitized by poly(I:C) in BRCA1-deficient breast cancer cells. Synergy score was higher in SUM149 due to lower sensitivity to olaparib in SUM149 than in HCC1937, but a similar inhibition effect of combination treatment in both cells. Furthermore, we evaluated the expression of downstream genes of antiviral mimicry innate immune response, including IFIT, OAS, IFN, and IFN-stimulated genes CCL5 and CXCL10, after further activating this signaling by combining with poly(I:C). These genes showed a remarkable increase upon combination treatment of PARPi and poly(I:C) (Fig. 5G). To further evaluate the activation of immune cells, we cultured Jurkat cells with supernatant from HCC1937 cells upon PARPi olaparib, poly(I:C), or combination treatment. We performed western blot of IFNγ after culturing with supernatant. The result showed that supernatant from these three groups significantly increased expression of IFNγ, indicating activation of Jurkat cells (Fig. S7B). Moreover, supernatant of the combination group further increased IFNγ level, which suggests a combination of olaparib and poly(I:C) could trigger a more profound immune response. Overall, our data reveal that BRCA1 deficiency decreases IRF3 in breast cancer cells, which induces resistance to PARPi, and the resistance can be overcome by combination with poly(I:C).

### Combination of PARPi and poly(I:C) enhances anti-tumor efficiency *in vivo*

**Figure 6.**
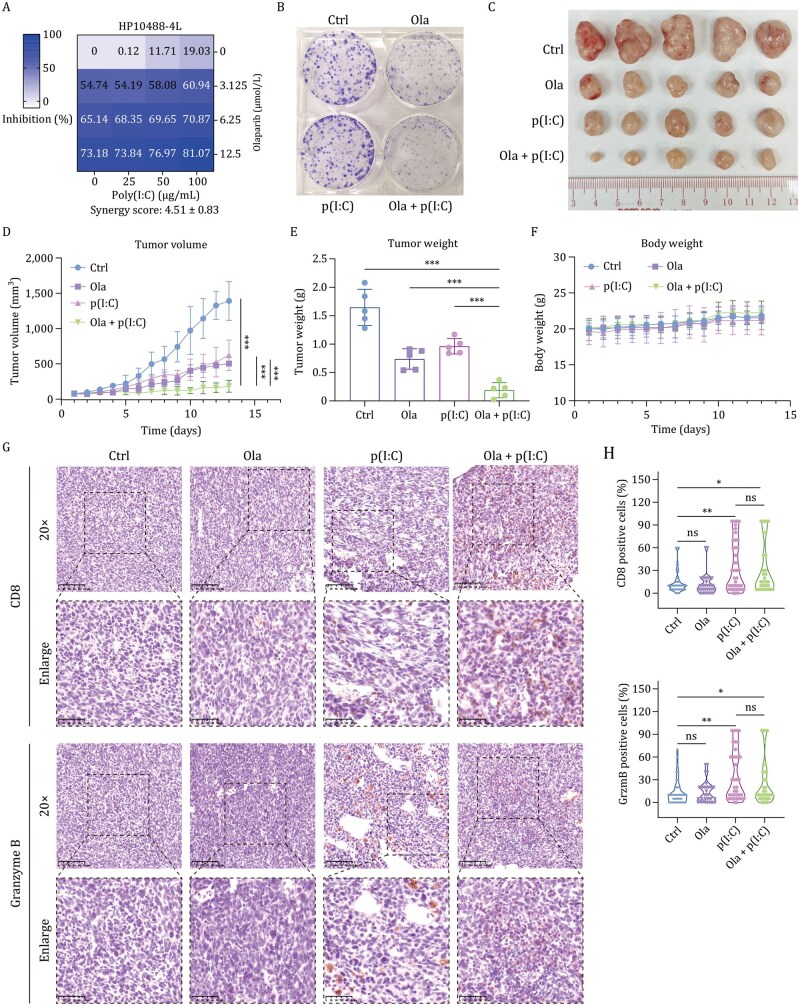
**Combination of PARPi and poly(I:C) enhances anti-tumor efficiency *in vivo***. (A) Synergy heatmap of combination treatment with olaparib and poly(I:C) in HP10488-4L Brca1-deficient breast cancer cells. Synergy score was calculated via the SynergyFinder web tool using the HSA method. (B) Colony formation of HP10488-4L cells upon olaparib (10 µmol/L) and poly(I:C) (25 µg/mL) single drug or combined drug treatment. (C–F) HP10488-4L cells were injected into the right mammary fat pads of female FVB mice. Then administered with 10 mg/kg poly(I:C) and 50 mg/kg olaparib every other day by intraperitoneal injection. Tumor volume (D) and body weight (F) were recorded every day. Tumors were excised (C) and weighed (E) at the endpoint. Data were presented as mean ± SD. ns, not significant; *P < 0.05; **P < 0.01; ***P < 0.001 (two-way ANOVA, *n *= 3). (G) Representative images of CD8 and granzyme B immunohistochemistry staining in the tumor tissue after olaparib, poly(I:C), or combination treatment. Scale bar: 100 µm in 20× images; 50 µm in enlarged images. (H) Percentage of positive staining cells of CD8 and granzyme B immunohistochemistry in G (ns, not significant; *P < 0.05; **P < 0.01; ***P < 0.001, Mann–Whitney *U* test).

Before evaluation *in vivo*, we tested synergy effect *in vitro* in the mouse Brca1-deficient breast cancer cell line HP10488-4L. HP10488-4L cell line derived from a mammary tumor of a mouse that carries a mammary-specific knockout Brca1 (*Brca1^co/co^*; *Cre-MMTV*) in FVB background (Fig. S8A) (Xu et al., 1999a). The synergy score in HP10488-4L indicated that the two-drug combination had additive effect (Fig. 6A). The colony formation assay showed similar results that combination treatment reduced more colony formation compared to control and single drug treatment in HP10488-4L (Fig. 6B). To examine the combination effect of PARPi and poly(I:C) *in vivo*, we established mouse breast cancer allograft tumor by injecting 1 × 10^6^ HP10488-4L cells into mammary fat pads of 6-week-old female FVB mice. We then treated the mice with single drug therapy or a combination of both drugs and monitored tumor growth. 10 mg/kg poly(I:C) and 50 mg/kg olaparib were administered intraperitoneally to mice every other day. PARPi together with poly(I:C) significantly inhibited tumor growth compared to control and single drug-treated tumors (Fig. 6C and 6D). Two-drug-treated mice also exhibited a significant reduction in tumor weight without an obvious change in body weight (Fig. 6E and 6F). Together, mouse studies indicate that a combination of PARPi and poly(I:C) can enhance anti-tumor efficiency *in vivo*, and poly(I:C) can be an immune adjuvant agent for PARPi treatment in BRCA1-deficient breast cancer therapies. Due to the combination of PARPi and poly(I:C) inducing innate immune response and expression of CCL5, CXCL10, and IFN, which are associated with CD8^+^ T cell infiltration and activation (Ozga et al., 2021), we performed CD8 and granzyme B immunohistochemistry (IHC) on the above tumors. As shown in Fig. 6G and 6H, CD8 and granzyme B-positive cells were significantly increased in poly(I:C) single-drug and two-drug treatment groups, suggesting that PARPi and poly(I:C) treatment further activate the adaptive immune response. We also performed IF of cleaved caspase-3 and CD8. Olaparib, poly(I:C), and combination treatment tumors showed an increase of cleaved caspase-3, indicating activation of the apoptotic pathway (Fig. S8B). Moreover, cleaved caspase-3 and CD8 overlapped in some areas, which suggests CD8^+^ T cells might promote apoptosis upon poly(I:C) and combination treatment.

## Discussion

BRCA1 carriers are at high risk of getting cancers, especially breast cancer. BRCA1-deficient breast cancers tend to develop into triple-negative breast cancers (TNBCs) and basal-like subtypes. These subtypes of patients are usually more malignant with poor prognosis. Treatment of BRCA1-deficient patients remains a clinical challenge due to limited therapy strategies (Lee et al., 2011). PARPi are the only targeted therapy for BRCA1-deficient patients based on the rationale of synthetic lethality. Olaparib is the first FDA-approved PARPi, followed by other PARPi, including niraparib, rucaparib, talazoparib, and veliparib. PARPi has been approved for BRCA-deficient breast cancer, platinum-responsive advanced ovarian cancer, BRCA-deficient pancreatic cancer, and HR-deficient prostate cancer. Researchers are still trying to expand indications, along with increasing knowledge about PARPi. Here, we used a functional proteomics approach to further understand the mechanism of action of PARPi. We found PARPi dysregulates the function of the spliceosome, which induces accumulation of dsRNA and triggers antiviral mimicry innate immune response. However, the response is compromised in BRCA1-deficient breast ­cancer cells.

Recently, immune signaling has been characterized in consequence of unstable genome in BRCA1/2-deficient cancers, especially after DNA damage drugs and radiation therapies (Ding et al., 2018; Pantelidou et al., 2019; Shen et al., 2019). DsDNA then releases into the cytoplasm and triggers the cGAS-STING pathway. Chemokines and cytokines like IFN, CCL5, and CXCL10 are secreted via tumor cell intrinsic innate immune response, which further activates immune cells in the tumor microenvironment. Apparently, to avoid clearing by effective immune surveillance, cancer cells with genomic instability need to raise mechanisms to overcome consequence of innate immune signaling. Accumulating evidence indicates that multiple adaptive mechanisms facilitate immune escape, which will compromise drug response and result in resistance to DNA damage drugs, including PARPi. For instance, upregulation of cytoplasmic nucleases such as TREX1 and RNase H2 promotes degradation of cytoplasmic DNA or RNA and represses the following immune response (Mackenzie et al., 2016; Yang et al., 2007). Another mechanism study shows mutant p53 proteins can competitively bind with TBK1 and prevent TBK1 binding to IRF3 and STING, leading to inactivation of innate immune signaling (Ghosh et al., 2021). Besides, MYC attenuates innate immune response and promotes tumor immune escape through suppression of signaling regulators like STING, IRF5, IRF7, STAT1, and STAT2 (Lee et al., 2022b; ZimmerLi et al., 2022). Some studies also suggest that BRCA1 deficiency facilitates immunosuppression (Li et al., 2022; Samstein et al., 2021). In our study, we show a novel mechanism that BRCA1 deficiency downregulates IRF3 and thereby suppresses innate immune response in breast cancer cells. Specifically, we discovered PARPi treatment causes accumulation of dsRNA and then activation of MAVS, but the downstream dsRNA antiviral mimicry innate immune response is dramatically lower in BRCA1-deficient breast cancer cells. Furthermore, we reveal that the response is compromised in BRCA1-deficient cells by repression of IRF3.

The crosslink of DDR and innate immune response was widely identified. The connection between these two signaling is dsDNA and dsRNA. DNA damage causes the formation of a micronuclei envelope, which will be disrupted in the cytosol (Mackenzie et al., 2017). dsDNA is then released and recognized by the dsDNA sensor cGAS. Finally, cGAS-STING innate immune signaling is activated. Additionally, dsRNA-MAVS signaling is also crucial in the innate immune response. Some DNA-damage-drug-treatment and radiation can cause accumulation of dsRNA (Chiappinelli et al., 2015; Guo et al., 2022; Murayama et al., 2024; Patel et al., 2022). Several mechanisms for the accumulation of dsRNA have been identified. AT-rich DNA fragments can be transcribed to dsRNA by RNA polymerase III and accumulate in the cytosol (Feng et al., 2020). Endogenous retroviral elements are reported as one of the sources of dsRNA (Murayama et al., 2024). Moreover, ­perturbation of RNA splicing by spliceosome inhibitors induces dsRNA accumulation (Bowling et al., 2021). Our results reveal a novel mechanism in which the disturbance of splicing machinery homeostasis facilitates dsRNA accumulation. PARP1 binds to splicing factor SF3B1, RNA, and chromatin to mediate co-transcriptional splicing (Matveeva et al., 2019; Melikishvili et al., 2017; Panzeri et al., 2023). Our study on breast cancer demonstrates PARPi treatment enhances the interaction of PARP1 with SF3B1 and RNAPII with SF3B1, resulting in dysregulation of RNA splicing. Consequently, PARPi induces differential alternative spliced events and dsRNA accumulation.

Currently, PARPi are mainly approved for HR-deficient cancer patients due to a specific DNA damage repair defective context, such as BRCA1/2-deficient breast cancer, BRCA1/2-deficient pancreatic cancer, HR-deficient prostate cancer, etc. Along with further understanding the mechanism of action of PARPi and resistance, the benefited population of patients has been extended. In 2018, the indications were expanded to completely or partially platinum response ovarian cancer patients regardless of BRCA status (Ledermann et al., 2014). In addition to DDR, the efficacy of PARPi depends on the immune stimulatory effect (Pantelidou et al., 2019). Accordingly, the combination of PARPi and anti-PD1 or PD-L1 antibody is explored in clinical studies (Kim et al., 2023; Lampert et al., 2020). Our studies reveal that the efficacy of PARPi is sensitized by further activating dsRNA innate immune signaling via combination treatment with poly(I:C). Finally, we show that the combination of PARPi and poly(I:C) enhanced anti-tumor efficiency *in vivo*. Our study reveals that PARPi and poly(I:C) have a synergy effect in BRCA1-deficient breast cancer, which is a potential strategy to treat BRCA1-deficient tumors.

In summary, we investigate a novel function of BRCA1 that BRCA1 increases innate immune response by upregulation of IRF3, while BRCA1 deficiency results in lower response of this signaling via repressing IRF3. We also reveal detailed mechanistic results about how BRCA1 and PARP1 crosstalk with the innate immune response. More importantly, we provide a novel combination strategy, PARPi and poly(I:C), to overcome intrinsic resistance caused by low response of innate immune signaling in BRCA1-deficient breast cancers.

## Supplementary Material

pwaf104_Supplementary_Data

## Data Availability

RNA-seq datasets in this study are available in the NCBI GEO database under accession number GSE305504. Proteomics data from mass spectrometry have been deposited to the ProteomeXchange Consortium (proteomecentral.proteomexchange.org) via the iProX partner repository (Chen et al., 2022; Ma et al., 2019) with the dataset identifier PXD067930.

## Abbreviations

A3SS, alternative 3ʹ splice sites; A5SS, alternative 5ʹ splice sites; BRCA1, breast cancer-associated gene 1; BER, base-excision repair; cGAS, cyclic GMP/AMP synthase; DsRNA, double strand RNA; DsDNA, double strand DNA; DSBs, DNA double strand breaks; DDR, DNA damage response; HR, homologous recombination; IRF3, interferon regulatory factor 3; IFIT, interferon induced protein with tetratricopeptide repeats; IFN, interferon; LiP-MS, limited proteolysis-mass spectrometry; MAVS, mitochondrial antiviral signaling protein; MXE, mutually exclusive exons; MMEJ, microhomology mediated end joining; NHEJ, nonhomologous DNA end joining; OAS, 2ʹ-5ʹ oligoadenylate synthetases; PARPi, poly (ADP-ribose) polymerase 1 inhibitor; PARylation, poly ADP-ribosylation; Poly(I:C), polyinosinic-polycytidylic acid; PK, proteinase K; RI, retained intron; RNAPII, RNA polymerase II; SSBs, single strand DNA breaks; SE, skipped exon; SDD-AGE, semi-denaturing detergent agarose gel electrophoresis; STING, stimulator of interferon genes; TBK1, TANK-binding kinase 1.
